# “Sympathy” vs.“Empathy”: Comparing experiences of I2Audits and disability simulations

**DOI:** 10.3389/fresc.2022.876099

**Published:** 2022-09-05

**Authors:** Emily C. Hicks, Meg A. Traci, Karin Korb

**Affiliations:** ^1^ Rural Institute for Inclusive Communities, University of Montana, Missoula, MT, United States; ^2^ Karin Korb LLC, Ft. Lauderdale, FL, United States

**Keywords:** i2Audit, disability simulation, disability, rural, stigma, built environment

## Abstract

People with disability often experience stigma and discrimination, and people with disability in rural areas may experience these at higher rates. Additionally, people with disability in rural areas may have fewer opportunities for physical and social participation due to barriers in the built environment. Activities such as disability simulations and inclusive, interdisciplinary community planning workshops (i.e., I2Audits) seek to draw awareness to and address these problematic experiences. The present study used thematic analysis from qualitative research to examine the advantages and disadvantages of using disability simulations and I2Audits in rural communities. Findings suggest that disability simulations increase stigmatization, lead to feelings of embarrassment and discomfort, and do not capture the experiences of people with disability. On the other hand, I2Audits lead to meaningful environmental changes, create feelings of empowerment, and center the lived experiences of people with disability within a bio-psycho-social model of disability. Results suggest that not only can I2Audits be a powerful tool to draw attention to physical barriers that people with disability face, but they also draw attention to the multi-level changes needed to increase opportunities for participation and address sources of stigma and discrimination in rural areas.

## Introduction

According to the Centers for Disease Control and Prevention (CDC), one in four (61 million) adults in America report having a disability [as cited in (1)]. The World Health Organization and World Bank reported that 15% of the world's population, or one billion people, experience some form of disability (2). Further, approximately 60 million Americans live in rural areas (3), and of adults in rural areas, one-third report a disability (4). Of note, these numbers may be underestimated due to lack of disability disclosure due to disability stereotypes and discrimination, as people with disability^1^ experience noteworthy challenges, including inaccessible environments, stigma, and negative attitudes (5).

Stigma first began to be looked at in modern times by Erving Goffman, and he defined stigma as an “attribute that is deeply discrediting” (6, *p*.3). It has since been defined in other ways, such as “the process by which a society bestows its own negative meaning on the behaviors, signs, or attributes of an individual'‘ (7, p. 39), and “social devaluation or the potential for negative treatment” (8, p. 3). Although these definitions may seem abstract, they have real-world implications for millions of people. For example, a recent field study found that fictional applicants with disabilities received 26% fewer statements of interest from employers compared with fictional applicants without disabilities, with little difference between applicants other than disability status (9). Additionally, the Royal Mencap Society reported that children with special education needs (SEN) are twice as likely to be bullied as children without any SEN (10). Furthermore, only 6% of adults with a learning disability were in paid employment that was reported to local authorities in 2017/2018 in England, compared with 76% of the general population ages 16 to 64 (10). Studies have found that experiencing stigmatization is related to more depressive symptoms and decreased emotional well-being in persons who self-identify as having a disability (11, 12). Additionally, greater depression severity was found to be a statistically significant predictor of perceived stigma (13). So, the relationship between stigma and depression may be circular, creating negative cycles of poor social experiences and mental health outcomes in persons with disabilities and chronic illnesses.

People with disability in rural areas may face increased stigmatization and discrimination (14). Qualitative research examining the lived experiences of people with disabilities in rural areas indicates that individuals experience increased isolation, violence, and social exclusion (14). Further, people with disability in rural areas experience barriers to healthcare due to accessibility concerns. There are significant differences in healthcare access for those in rural vs. urban areas, with individuals in rural areas facing a lack of public transportation, fewer health services, and cultural and financial concerns (15). For example, in rural areas, healthcare professionals may also be friends and neighbors, which may hamper a person's level of comfort with seeking services and sharing personal, medical information (15). Additionally, finances may prohibit treatment-seeking, as research has noted an inequality in health care coverage between urban and rural areas such that rural areas tend to have higher numbers of uninsured individuals than urban areas (16). This factor combined with higher rates of poverty in rural areas (17) leads to multiple financial barriers in accessing care. For people with disability living in rural areas, the financial concerns of living in a rural place may be compounded by disability status, as research has found statistically positive relationships between disability and poverty (18). Thus, access to services in rural areas may be hindered on multiple levels, which contributes to people in rural areas experiencing poorer physical and mental health (19). For example, people residing in rural areas are more likely to experience chronic conditions, activity limitations, and are 1.5 times more likely than those in urban areas to rate their physical health as “fair to poor” (19).

Social participation and perceived isolation have also been linked to the health of people with disability (20). Research suggests that decreased social participation and increased isolation is related to poorer health and less satisfaction for people with disability (20). Objective measurements have indicated that rural areas tend to be less accessible than urban areas (21). This is significant as community environments with poor accessibility lead to less opportunities for social participation.

Barriers to social participation for people with disability in rural areas may also be attributed to lack of transportation. Data from the Rehabilitation Services Administration (RSA) suggests that people with disability in rural areas receive less transportation services than people with disability in urban areas (22). Previous research has cited inadequate public transportation to be a barrier to social participation, especially for those in rural communities and those with mobility issues (23). Transport systems not only include the availability of public transportation, but also the physical characteristics of environments, planning processes, design, and policies that allow people to move from place to place (24). The Association of Programs for Rural Independent Living (APRIL) describes transportation as the “systems, services, vehicles, routes, stops, programs, and all other aspects of transportation” (25). Accessible transport systems have been linked to well-being (24). Thus, it is important to consider the relationships between transport systems, social participation, isolation and exclusion, accessibility of the environment, and health and well-being for people with disability in rural areas.

The recognition that people with disability in rural areas experience barriers to health services (15), social participation (20), and transportation (22) due to physical, sociocultural, and sociopolitical obstacles has led to a variety of efforts aimed at drawing attention to these critical issues. One effort that has frequently been used is a disability simulation. Disability simulations are “interactive role-playing experiences to improve disability attitudes and increase understanding” (26, p. 324). These simulations may ask participants to wear blindfolds or glasses to mimic low vision, earplugs to approximate hearing loss, or go through their day in a wheelchair to imitate the day of someone with paraplegia. While these exercises are likely engaging for the participants, little research has examined the effectiveness of these traditional disability simulations to improve attitudes and increase understanding (27). A meta-analysis examining the effectiveness of disability simulations suggests that there is a lack of research regarding effectiveness, as much of the research surrounding simulations describes steps to hosting a disability simulation (27). A recent study examined this issue and found that across two experiments, students who completed low vision, hearing impairment, dyslexia, or mobility impairment simulations felt more confused, embarrassed, helpless, and susceptible to becoming disabled after the simulation compared to baseline (26). Additionally, the study found that while empathetic concern increased in both studies, participants ultimately expressed greater discomfort about interacting with persons with disabilities following the simulation (26). Therefore, there is some evidence that traditional disability simulations do not fulfill their stated goals of improving attitudes and increasing understanding. This idea was supported by a meta-analysis examining ten disability simulations that suggested, based on effect sizes, that there was little evidence to suggest that disability simulations effectively improve attitudes towards people with disability (27). In fact, some researchers have noted that disability simulations not only fail to show the reality of disabilities, but they also actually perpetuate the stereotypes of incompetence and dependency (26). These simulations have been critiqued as misleading participants to think that the source of disadvantages is the person with the disability, while ignoring environmental barriers and government policies that are discriminatory and stigmatizing towards some people (26), leading to harmful effects. Other critiques of disability simulations suggest that the simulations may ultimately lead to discrimination due to reinforced stereotypes (28). It should be noted that while some studies have suggested that disability simulations lead to harmful outcomes (26), others have demonstrated that while disability simulations are ineffective, they are not harmful (27). Still other studies have found mixed results of disability simulations, with one study noting both positive attitude changes and no attitude changes following a simulation (29).

Notably, a meta-analysis of disability simulations explored the factors that may lead to improved attitudes towards people with disability. Results indicated that interaction with people with disability was most effective (27). In fact, this interaction was described as “an essential component of attempts to change attitudes or behaviors related to people with disabilities” (27, p. 76). This suggestion is supported by empirical research, which has indicated that students who interact with children with disability in classroom environments were more accepting of their peers with disability than children in classrooms without disability representation (30). In another study, nursing students engaged in a disability simulation in pairs, with one student acting as a “patient” and simulating hemiparesis, and the other student acting as a “rehabilitation nurse” (31). Results of the study indicate that while all students had increased empathy scores after the simulation, the students acting as rehabilitation nurses had higher empathy scores post-simulation (31). Although disability was simulated in this study, the results suggest that higher empathic changes can occur when engaging with people with disability rather than pretending to have a disability. Ultimately, it has been suggested that interacting with people with disability may lead to greater acceptance and understanding than disability simulations (27, 28).

As an alternative approach to disability simulations, an inclusive, interdisciplinary model may be better suited to truly increase understanding, improve disability attitudes, and instigate institutional and government policy changes. Interdisciplinary systems are “teams or individuals that integrate information, data, techniques, tools, perspectives, concepts, and/or theories from two or more disciplines or bodies of specialized knowledge to advance fundamental understanding or to solve problems whose solutions are beyond the scope of a single discipline or area of research practice” (32, p. 2). Inclusive, interdisciplinary models have been found to have benefits for students in both primary (33) and secondary education (34), enhance professional growth and trust among colleagues (35), and improve patient outcomes in healthcare settings (36). Due to the promising findings from inclusive, interdisciplinary models found across community settings, the Rural Institute at the University of Montana developed the Inclusive Interdisciplinary Audit Toolkit (I2Audit) in 2018.

The I2Audit can be utilized to assess environmental needs and “work together to find equitable policy, systems, and environmental (PSE) solutions” (37). Walk/move audits are facilitated group explorations of an area to examine its support of physical activity and active transportation. The I2Audit builds on traditional walk/move audits and is inclusive in that people with disability lead and are decision-makers in the planning, implementation, and evaluation of the audit. This model highlights that audit events should facilitate acts of shared discovery, and not simply showcase experts telling others what they should be experiencing. The I2Audit also differs from traditional walk/move audits in that it is interdisciplinary and encourages teams to consist of people with disability, and representatives from disability advocacy, public health, planning and land use, and engineering and infrastructure systems. The team evaluates an area's sidewalks, bike lanes, curb ramps, and transit options for site design, safety, and accessibility. Since its inception, sixteen communities in a rural state have used the I2Audit and established 23 policies and plans for built environments focused on physical activity, including 11 inclusive complete streets policies (37). The I2Audit model presents a promising alternative to traditional disability simulations due to its focus on the people with disabilities, rather than the disability itself. This model may be particularly useful in rural communities, as research suggests that rural areas tend to have limited built environment features that promote active living and active transportation (38). These limitations lead to fewer opportunities for physical activity (38), less access to health care services (15), reduced social participation (20), and decreased well-being (23). Additionally, people with disabilities are often not included in rural development interventions, leading to social exclusion (5), despite research noting that people with disability are active, effective leaders and participants in projects aimed at developing accessible rural areas (39). I2Audits create opportunities for people with disability and other community members to work alongside each other to identify barriers and develop solutions to improve features of the built environment that limit healthy lifestyles for people with disability in rural areas. While the I2Audit is designed to address concerns regarding social participation, the I2Audit is also used to address concerns surrounding stigma and discrimination. Of importance, the I2Audit is purposeful in pairing people with and without disability to explore their community, and interaction with people with disability has been cited as a key factor in instigating greater acceptance of people with disability (27).

## Methods

Given the relative lack of research regarding disability simulations, limited evidence noting the problematic outcomes of disability simulations, and the promising nature of inclusive, interdisciplinary models, the present study sought to gain insight into the advantages and disadvantages of disability simulations and I2Audits from community members who had participated in one or both activities in rural communities. Thus, a Participatory Action Research (PAR) modality was chosen, and the researchers sought insight from community members and stakeholders, including people with disability, state Department of Public Health and Human Services (DPHHS) representatives, and active community members. The researcher's original idea for the study was to conduct both a disability simulation and an I2Audit and obtain both objective and subjective data regarding the effectiveness of the interventions in improving attitudes toward people with disability. However, upon proposing the research design, group members strongly objected to the idea of conducting a disability simulation as part of the study. The group suggested that given the research that disability simulations may be harmful (i.e., 26), it would be unethical to conduct such a simulation. Thus, the research objective was altered. Rather than conducting both a disability simulation and an I2Audit to compare effectiveness, the researchers aimed to explicitly describe the concerns with disability simulations and how I2Audits may be similar or dissimilar to disability simulations. The PAR group members approved this approach and felt that this methodology may delineate between helpful and unhelpful practices that aim to improve attitudes toward people with disability.

As in all research, it is helpful to understand the positionality of the researchers as this influences the lens through which the study is conducted, and the results understood. The first author is a non-Disabled, American Indian, cisgender woman, and a U.S. scholar. She is a member of tribal communities and works within the local community with people with disability as a researcher and mental health clinician. The second author is non-Disabled, White, cisgender woman, and an academic researcher with decades of experience working with people with disability and advocacy organizations using a Disability-led participatory approach and methods. The third author is a Disabled, White, cisgender woman and Disability thought leader and Diversity, Equity and Inclusion advocate. Authors had discussions with each other, as well as with people with disability and community leaders, to ensure the study was guided by cultural knowledge and expertise.

### Sample

The sample consisted of 12 participants residing in a large, rural state. Participants included community members, disability advocates, and university students who had participated in a disability simulation, I2Audit, or both. Convenience sampling was used, and a recruitment email was distributed by the PAR group. The email was sent to disability advocates, community members, and stakeholders who were known to have either participated in a disability simulation or I2Audit. Participants were excluded from the study if they were under 18-years-old or had not participated in either a disability simulation or an I2Audit.

### Procedure

A targeted approach was used, and a qualitative survey was emailed to participants. Participants were asked to describe their previous experiences with disability simulations and I2Audits. Additionally, they were asked to note how they found the two experiences to be similar and/or different if they had participated in both activities. Finally, they were asked to list any policies, systems, or environmental changes that had occurred in their communities as a result of I2Audits. Specific questions included, but were not limited to: (1) Please describe your past experiences with I2Audits, (2) If you've participated in a disability simulation awareness training, what do you perceive as the major differences between disability simulation trainings and I2Audits, (3) Do you feel that disability simulation trainings or I2Audits are more effective for community understandings of the experiences of people with disabilities, and (4) Please describe any policy, systems, environmental or program changes that have occurred as a result of an I2Audit that you participated in. Participants were also given space to detail other information they would like to share about disability simulations and/or I2Audits and their effects.

The PAR group advised the recruitment, development, outcomes, and dissemination phases of the study. In order to honor the words of participants, a qualitative descriptive methodology was used rather than a method that forces categorical responses. Descriptive methodology is especially important when the research concerns underrepresented groups in research, including people with disability, as researchers have historically taken advantage of marginalized communities. Additionally, qualitative research is often useful in understudied research areas, including the topic of the present study. To best respect and understand participant's responses, a qualitative study protocol that uses verbatim responses was used. Authors had conversations with each other and members of the PAR group to determine overarching themes based on the participant's responses. While this innately requires a level of data interpretation, qualitative methodologies allow for this interpretation to occur while considering cultural, societal, and contextual factors. For example, some participants identified as having a disability, while others did not. Disability status may influence people's experiences of disability simulations and I2Audits – a contextual factor that is important for data interpretation.

## Results

### Participant demographics

The majority of the participants identified as White (*n* = 11), female (*n* = 8), and had some college education (*n* = 12). Five of the participants identified as having a disability (*n* = 5). All participants resided in a large, rural state at the time of the study. All participants had professional and personal experiences working with individuals and community organizations in rural counties in the state as well. The majority of participants had participated in both a disability simulation and an I2Audit (*n* = 7), with three participants having only participated in a disability simulation (*n* = 3), and two participants having only participated in an I2Audit (*n* = 2). Questions about the specific disability simulation or I2Audit the participant had participated in were not asked; however, several participants disclosed this information in their responses to other questions. Three participants described participating in a disability simulation meant to mimic speech impairment, and four participants stated the city of the I2Audit that they participated in. The disclosed cities had populations of 34 to 75 thousand people and were located in a rural state.

### “It made me feel flawed”: Disability simulations ignore environmental barriers

The first theme that emerged surrounded the idea that disability simulations ignore environmental barriers. One participant noted, “Instead of learning about the social and environmental barriers experienced by people with disability and having that community provide solutions, the simulations just have us learn that it is harder to move around the world.” This participant highlights the stigma that people with disability face by people assuming that difficulties can be attributed to the individual, rather than access concerns. A second participant stated, “It [disability simulation] made me feel flawed. I just felt like I couldn’t do anything right.” Again, this participant draws attention to the individual stigma that one may experience, rather than considering broader, environmental barriers. A final participant described, “They [disability simulation] made it impossible to succeed. I think with time and practice I could learn to live with it [dyslexia], but it was so short that we all just felt annoyed.” This participant highlights the idea that a person can live a full life with environmental support but managing discrimination can be difficult without this support.

### “A different mindset”: I2Audits provide a variety of perspectives

A second theme that emerged was that I2Audits offer a variety of perspectives. One participant noted, “[It is] very helpful to experience an area with a different mindset.” This quote describes that the participant was able to listen to and learn from someone else's perspective on the built environment of a space. This idea was corroborated by another participant who stated, “It allows all participants to have a voice and not be overshadowed.” In addition to highlighting shared perspectives, this quote underscores that each participant's knowledge is considered equally important. Another participant expressed, “I always learn a lot and gain a different perspective…It is good to hear a variety of perspectives.” This participant appears to have engaged in multiple I2Audits and has gained different knowledge with each experience. Finally, a participant said, “Everyone had a chance for their voices to be heard.” This quote draws attention to the idea that there is no expert in I2Audits, but rather, each perspective is considered valuable and meaningful.

### “Instead of increasing my skill…it increased…fear”: Embarrassing vs. empowering

Participants who had participated in both disability simulations and I2Audits tended to describe the disability simulations as uncomfortable or embarrassing, while describing the I2Audits as empowering and helpful. For example, one participant noted, “An I2Audit includes people with disability to share their lived experience and influence change through their perspective. The disability simulations I have been a part of do not involve people with disability…There is little context put to these simulations and therefore sympathy, instead of empathy and partnerships, is developed. The I2 audit fosters the latter and gives the participants a new perspective and skills to make change. Therefore, systems change is more likely to occur on that specific project and in future projects.” This participant notes that disability simulations, while positive in their intentions, may lead to pity rather than empathy. A second participant described, “I used to think those [disability simulations] were good tools but since doing I2WALK [I2Audit] trainings where people with a variety of mobility, vision, or cognitive challenges are the actual leaders and can share their life experience and people can see how they need to navigate a flawed environment… I feel the I2WALK [I2Audit] audits are more powerful.” Again, this participant draws attention to the idea that while both disability simulations and I2Audits attempt to bring positive change for the disability community, the I2Audits do so in a way that empowers the disability community. A final participant stated, “The other simulation I was a part of required me to wear earplugs for a day and not talk. I was embarrassed to do so. I don't think I was embarrassed to have a “disability” but rather that I had to pretend to do so… Instead of increasing my skill to support people with disability, it increased sympathy and fear. I have never forgotten these experiences. At the time I didn't know why they felt wrong, but I am now glad to have the skills to include people with disability, to amplify their voices, and promote audits that increase individual, social, and environmental change in our communities.” This participant describes that disability simulations were uncomfortable, even if they were unsure why, and that learning how to be alongside people with disabilities, rather than in place of them, was an empowering experience.

### “Tool for…community engagement”: Engaging with people with disability leads to meaningful interactions and environmental changes

The fourth and final theme that emerged was that engaging with people with disability led to meaningful interactions and changes in the built environment. I2Audits were described by participants as a “change agent,” and participants identified specific shifts in the infrastructure of their towns as a result of I2Audits. For example, one participant stated, “[City name's] wayfinding system was created from the I2Walk/Move audits [I2Audit], too. I use those signs weekly.” Another participant remarked, “The uptown [City name] Master Plan is an extension of many years of work inspired by walk audits. Many new bus stops and curb extensions have come from walk audits [I2Audits].” Another city's downtown master plan was also described as being informed by I2Audits. Finally, a participant remarked, “I think they [I2Audits] are a great tool for general community engagement, educating policymakers and staff, and engaging people with disabilities and learning from their life experiences for the betterment of all of us.” This final quote highlights that not only do I2Audits create awareness around disability, but they also lead to environmental changes and further the education of community members and leaders.

## Discussion

People with disability experience stigmatization and decreased social participation that negatively impact both physical and mental health. Thus, efforts have been made to improve attitudes and understanding toward people with disability; however, some efforts that have been taken (e.g., disability simulations) have been found to be ineffective and possibly harmful. There is a need for interventions that improve attitudes toward people with disability, as well as interventions that may increase social participation and active community engagement. It has been suggested that interaction with people with disability may be more effective than disability simulations. Based on past research, Inclusive, Interdisciplinary Audits (I2Audits) sought to fill the gap in increasing interaction with people with disability in order to improve disability attitudes and understanding.

Overall, results suggested that participants found disability simulations to increase fear, frustration, and embarrassment. In contrast, participants found I2Audits to allow for sharing of perspectives, increased empathy, and learning from the experiences of people with disability. Participants identified multiple environmental changes that occurred as a result of I2Audits, including changes to bus stops, curb extensions, and wayfinding systems. Of importance, these changes occurred in rural communities. While previous research has documented the limited infrastructure changes of rural built environments (38), I2Audits led to meaningful improvements to the built environment in rural areas, increasing opportunities for physical and social engagement for people with disability in rural places.

It is common during I2Audit planning processes for new team members to suggest that traditional elements of disability simulations such as wheelchairs or blindfolds be added to the audit. It also is common for I2Audit participants to suggest adding disability simulations as follow up activities. These findings can support I2Audit teams to facilitate difficult conversations about negative experiences associated with traditional disability simulations, refocusing participants towards community changes that will better support people with disabilities.

Given the past research indicating ineffective and potentially problematic outcomes of disability simulations, combined with promising findings regarding I2Audits, communities should be encouraged to discontinue disability simulations and implement other interventions, such as I2Audits. A major difference between disability simulations and I2Audits is that I2Audits center the lived experiences of people with disability, while disability simulations mimic the experiences of people with disability; however, this mimicry does not lead to the desired outcomes of (1) increased recognition that there are layers of challenges in communities that limit and oppress people with disability; (2) increased engagement with people with disability and disability organizations; (3) prioritizing solutions developed in the disability community that, from a universal design perspective, can benefit all; and (4) valuing the lived experiences of people with disability and centering their subject matter expertise in community planning and decision-making. Allowing people with disability to lead the discussion on needed environmental and societal changes leads to improved outcomes and is in line with Participatory Action Research principles. Thus, interventions that prioritize having people with disability in leadership and decision-making roles will likely be more effective in meeting research and community goals.

### Limitations, future research, and recommendations

The primary limitation of the present study was that this was a convenience sample and the use of online mechanisms to gather qualitative data. Though participants had information or direct experience with both interventions, participants varied in their exposures to the interventions. Additionally, while participants provided detailed responses, the researchers had many follow-up questions that could have been answered more fully through an interview process. Future studies using qualitative interviews would be useful to clarify the information obtained in the present study. Additional limitations of the study include the small sample size and the use of subjective data. Future research should use objective measures to assess for differences in empathy, stereotype beliefs, and attitudes towards people with disability when examining the effectiveness of I2Audits.

The present research can be used to inform future qualitative research measurement selection when comparing simulations and audits, where measures are appropriate and culturally sensitive. Future research could evaluate the short- and long-term impacts of disability simulations vs. I2Audits on rural community group composition and planning priorities. Findings suggest that disability simulations that do not center the lived experiences of people with disabilities are an outdated, and generally problematic, exercise. This observation needs to be taken into consideration for future applications of disability simulation models such as experiencing wheelchair basketball and adaptive sports. Educators and researchers should instead consider using alternative activities, such as I2Audits, that highlight listening to and learning from people with disabilities.

### Contributions to the field

The present study seeks to fill a gap regarding the advantages and disadvantages of using disability simulations and Inclusive, Interdisciplinary Audits (I2Audits) in rural communities. While both aim to increase awareness towards barriers that people with disability face, findings suggest that disability simulations do not capture the experiences of people with disability and instead lead to embarrassment and discomfort and perpetuate stigmatization. On the other hand, I2Audits center the lived experiences of people with disability and lead to empowerment for people with disability and other community members. Additionally, I2Audits have led to positive built environment changes for people with disability in rural communities, creating additional opportunities for physical activity and social participation. These findings are particularly encouraging given past research noting the tendency to have limiting built environment features for people with disability in rural areas. Future research using objective data to compare outcomes between disability simulations and I2Audits in rural communities is needed.

## Data Availability

The original contributions presented in the study are included in the article/Supplementary files, further inquiries can be directed to the corresponding author/s.

## Footnotes

^1^The term people with disability (person first language) is used to reflect the current preferences of the disability community members with whom we engage on this work. Identity-first language is used in the positionality statements.
